# Establishment and functional evaluation of a two-stage mouse hepatocyte-derived organoid culture system

**DOI:** 10.3389/fcell.2026.1974273

**Published:** 2026-10-22

**Authors:** Yusi Chen, Yuting Shan, Li Tang

**Affiliations:** State Key Laboratory of Medical Proteomics, National Center for Protein Sciences (Beijing), Academy of Military Medical Sciences, Beijing, China

**Keywords:** hepatic maturation, hepatocyte expansion, *in vitro* model, metabolic zonation, mouse hepatocyte-derived organoid

## Abstract

**Background:**

Primary hepatocytes rapidly lose differentiated functions during prolonged *in vitro* culture, limiting their use in studies of liver metabolism and zonation. This study aimed to establish a two-stage mouse hepatocyte-derived organoid system that enables serial cell propagation followed by partial recovery of hepatic identity and metabolic function in three-dimensional culture.

**Methods:**

Primary mouse hepatocytes were first expanded in a two-dimensional transition and expansion medium and then transferred to low-attachment plates for three-dimensional culture in hepatocyte maturation medium. Cell growth, senescence, organoid formation, hepatic marker expression, bulk transcriptomic changes, albumin secretion, urea production, lipid and cholesterol metabolism, zonation-related responses, and post-cryopreservation recovery were evaluated. Statistical comparisons were performed using ordinary one-way or two-way analysis of variance, as appropriate.

**Results:**

The expansion medium supported serial propagation, maintained albumin expression, increased the proportion of Ki67-positive cells, and reduced senescence. A seeding density of 3 × 10^4^ cells per well produced compact and uniform organoids. Following transfer to 3D culture, organoids expressed albumin, E-cadherin, CYP2E1, and CK19. Bulk transcriptomic analysis showed strong cell-cycle activity in passage-2 cells, followed by partial recovery of hepatic metabolic programs during 3D culture, including cholesterol biosynthesis, the urea cycle, steroid biosynthesis, and bile-acid biosynthesis. Direct comparison with freshly isolated primary hepatocytes showed that albumin secretion remained lower than the primary hepatocytes level, whereas urea production increased during 3D culture and was not significantly different from PHCs by day 16. Exposure to 5% oxygen increased lipid-droplet accumulation. Glucose and insulin increased free and total cholesterol. Wnt/R-spondin increased the pericentral-associated marker glutamine synthetase, whereas ascorbic acid and bilirubin increased the periportal-associated marker CPS1. Early-passage cells retained organoid-forming capacity after cryopreservation and recovery.

**Conclusion:**

This two-stage culture system supports serial hepatocyte propagation followed by partial and function-specific recovery of hepatic identity and function in 3D culture. Although the organoids did not fully reproduce the mature primary hepatocyte state or physiological liver zonation, the system provides an experimentally accessible *in vitro* platform for investigating hepatocyte expansion, partial hepatic maturation, metabolic regulation, zonation-related responses, and microenvironment-dependent modulation of hepatocyte function.

## Introduction

The liver controls many metabolic and detoxification processes, including glucose metabolism, lipid synthesis and oxidation, amino-acid breakdown, urea production, bile-acid synthesis, plasma-protein secretion, and xenobiotic metabolism. These functions are arranged along the portal vein-to-central vein axis of the liver lobule. Oxygen, nutrients, hormones, and metabolic substrates change along this axis. These gradients produce periportal, midlobular, and pericentral hepatocyte states with different gene expression and metabolic activities (Aizarani et al., 2019; Ben-Moshe and Itzkovitz, 2019; Halpern et al., 2017; Jungermann and Katz, 1989; Martini et al., 2023; Samarah et al., 2025). An *in vitro* model that keeps these regional differences would help studies of normal liver function, injury, and disease.

Primary hepatocytes are widely used to study liver function, but they quickly lose polarity, liver gene expression, and metabolic activity in standard two-dimensional culture (Baker and Chen, 2012; Booth et al., 2012). Immortalized cell lines are easy to expand, but they do not reproduce many functions of normal hepatocytes. Liver organoids provide a three-dimensional model with cell-cell contact, tissue-like organization, and longer culture time (Booth et al., 2012; Huch and Koo, 2015; Lancaster and Knoblich, 2014). A main problem is that rapid expansion and mature liver function are difficult to maintain in the same system.

Early liver organoid studies showed that *Lgr5*-positive liver stem cells can be expanded for long periods (Huch et al., 2013). Human embryonic stem cell-derived hepatic organoids were later used to model alcoholic liver injury and liver repopulation (Wang et al., 2019). iPSC-derived liver buds and pluripotent stem cell organoids added vascular or multicellular features and were used for studies of tissue integration and steatohepatitis (Ouchi et al., 2019; Takebe et al., 2013). Recent models include mouse liver assembloids with periportal features, adult hepatocyte organoids with metabolic activity, and multi-zonal liver organoids (Dowbaj et al., 2025; Igarashi et al., 2025; Reza et al., 2025; Yuan et al., 2024).

These models provide increasingly sophisticated representations of liver architecture and function, but they also address different experimental goals from the present study. Here, we sought to establish a comparatively simple mouse hepatocyte-derived culture workflow in which cell expansion and subsequent hepatic functional recovery could be experimentally separated. Our approach uses two-dimensional expansion in TEM followed by low-attachment three-dimensional culture in HMM, allowing the proliferative state and subsequent partial hepatic maturation to be evaluated independently. The system was further designed for metabolic and environmental perturbation studies, including responses to oxygen, nutrients, and zonation-associated signals, as well as evaluation of post-cryopreservation organoid-forming capacity.

Patient-derived liver organoids can keep some features of the original tissue and have been used for disease modeling and drug screening (Ariñ et al., 2026; Broutier et al., 2017). Current systems still have limits in lobular maturation, immune and vascular components, and metabolic function (Booth et al., 2012; Dowbaj et al., 2025; Igarashi et al., 2025; Reza et al., 2025). A useful culture system should expand from a small number of starting cells, remain stable during passage, form three-dimensional organoids in a repeatable way, and recover major liver functions after 3D partial hepatic maturation. It should also respond to oxygen, nutrients, and signals linked to liver zonation.

We therefore developed a two-stage mouse hepatocyte-derived organoid system that separates two-dimensional cell expansion in TEM from subsequent three-dimensional partial hepatic maturation in HMM. We characterized the transition from freshly isolated hepatocytes to expanded cells and 3D hepatocyte-derived organoids using cellular, transcriptomic, and functional analyses, and further evaluated metabolic and zonation-related responses, serial passaging through P5, and post-cryopreservation organoid-forming capacity.

## Materials and methods

### Animal care and primary hepatocyte isolation

Male C57BL/6J mice, 8–10 weeks of age and 22–25 g in body weight, were obtained from Charles River (Beijing, China). Animals were housed in the specific pathogen-free facility at the Beijing Institute of Lifeomics. The room was maintained at 22 °C ± 2 °C and 50% ± 10% relative humidity with a 12-h light/12-h dark schedule. Standard chow and water were provided without restriction. The animal study was reviewed and approved by the Institutional Animal Care and Use Committee of the Beijing Institute of Lifeomics, Academy of Military Medical Sciences (approval no. NCPSB-20240110-07MT). All animal experiments were conducted in accordance with applicable institutional guidelines and local regulations.

Mice were anesthetized before liver perfusion. Perfusion Solution I was delivered through the portal vein at 3 mL/min, and the inferior vena cava was opened to provide an outlet. When the perfusate became clear, the liver was perfused with Perfusion Solution II at 3–4 mL/min for 5 min. The liver was then excised, washed in PBS, and transferred to DMEM containing 3% FBS. The tissue was gently dispersed to release hepatocytes. The cell suspension was filtered through a 70-μm mesh and centrifuged twice at 50 × g for 2 min at 4 °C using DMEM with 3% FBS. The resulting hepatocyte fraction was used for expansion and organoid experiments.

### Two-dimensional hepatocyte expansion and viability assay

Freshly isolated hepatocytes were plated on collagen-coated dishes at 2 × 10^4^ cells/cm^2^. Three culture conditions were compared: conventional medium (CM), LiverSup medium (M3C-02, xremed), and transition and expansion medium (TEM). CM was prepared with DMEM, 1% penicillin-streptomycin, 1% fetal bovine serum, and 1× GlutaMax. TEM used DMEM/F12 as the basal medium and contained N2, epidermal growth factor (20 ng/mL), hepatocyte growth factor (40 ng/mL), Y-27632 (10 μM), CHIR99021 (6 μM), sphingosine-1-phosphate (1 μM), lysophosphatidic acid (5 μM), and A83-01 (5 μM). Fresh medium was supplied every 48 h. Once cultures became confluent, cells were released with Accutase and replated at a 1:1 to 1:2 split ratio, usually once per week. Expansion was continued for three to five additional passages. Cell morphology was recorded with an inverted fluorescence microscope (Nikon Tie, Nikon).

Cell viability was followed with a CCK-8 assay. Hepatocytes were distributed into collagen-coated 96-well plates at 4–6 × 10^4^ cells in 100 μL medium per well. After a 4–6 h attachment period, fresh medium was added to remove non-adherent cells, and the planned treatments were started. For each reading, 10 μL of CCK-8 reagent was added to the culture. The plates were incubated for 2–3 h at 37 °C with 5% CO_2_ and protected from light. Optical density was measured at 450 nm, with 630 nm used as the reference. Relative viability (%) was calculated as (OD treatment - OD blank)/(OD control - OD blank) × 100.

### Three-dimensional organoid formation and maturation

To compare starting cell numbers, expanded cells were dispensed into round-bottom ultra-low-attachment 96-well plates at 0.5 × 10^4^, 1.0 × 10^4^, 2.0 × 10^4^, 3.0 × 10^4^, or 6.0 × 10^4^ cells per well. Each well contained 200 μL of a 1:1 mixture of TEM and HMM. Forty-eight hours later, 100 μL of the culture medium was removed and replaced with HMM. Spheroid assembly and morphology were followed from day 1 through day 8. The 3.0 × 10^4^-cell condition produced organoids with the most consistent size and intact shape, so this density was selected for later studies.

3D partial hepatic maturation was carried out in hepatocyte maturation medium (HMM). HMM consisted of DMEM/F12 with N2, DAPT (10 μM), oncostatin M (20 ng/mL), dexamethasone (10 μM), and SB431542 (10 μM). HMM was renewed every 48 h, and the maturation period lasted 12–21 days.

### Zonation-related induction

To assess responses to pericentral-associated Wnt signaling, Afamin-Wnt3A serum-free conditioned medium and R-spondin1-conditioned medium were added to HMM, with each factor present at 5 ng/mL. This concentration was selected based on a previously reported adult hepatocyte organoid culture system (Igarashi et al., 2025) and was used as an experimentally defined condition to modulate Wnt signaling rather than to reproduce an *in vivo* Wnt/R-spondin concentration gradient.

To assess responses to periportal-associated cues, bilirubin (1 mg/L) or ascorbic acid (0.01 mM) was added during the maturation phase. These treatment conditions were adopted from a previously reported multi-zonal liver organoid system (Reza et al., 2025) and were used to test zonation-related hepatocyte responses rather than to quantitatively reproduce physiological lobular gradients.

The same treatment was added again whenever the medium was renewed. Cultures were maintained at 37 °C in 5% CO_2_ and 95% air.

### Immunofluorescence staining and image analysis

The immunofluorescence panel included albumin (ALB), cytokeratin 19 (CK19), Ki67, E-cadherin, CYP2E1, glutamine synthetase (GS), and carbamoyl-phosphate synthetase 1 (CPS1). Hoechst was used for nuclear staining. The primary antibodies and their working dilutions are listed in Table 1.

**TABLE 1:** List of primary antibody.

Primary antibody	Supplier	Catalog number	Host species	Dilution
Albumin monoclonal antibody	Proteintech	66051-1-Ig	Mouse	1:300
CK19	Abcam	Ab52625	Rabbit	1:300
Ki67	Abclonal	A20018	Rabbit	1:300
Purified anti-mouse/human CD324 (E-cadherin)	BioLegend	147302	Rat	1:200
Cytochrome P450 2E1 (CYP2E1) rabbit mAb	Abclonal	A23030	Rabbit	1:300
GS	Abcam	AB49873	Rabbit	1:300
Anti-CPS1 antibody	Abcam	Ab129076	Rabbit	1:300

For two-dimensional samples, ethanol-treated coverslips were placed in 24-well plates and seeded with 5 × 10^4^ cells per well. When the cultures reached the required density, cells were rinsed in PBS and fixed with 4% paraformaldehyde. Permeabilization was performed with 0.3% Triton X-100, followed by blocking in PBS containing 5% goat serum. Samples were exposed to primary antibodies overnight. Fluorescent secondary antibodies were then applied for 2 h, followed by Hoechst staining and imaging.

Organoids were fixed in 4% PFA for 15 min at 4 °C, rinsed in PBS, and placed in chilled OWB buffer for a 15-min blocking step. Primary-antibody staining was carried out overnight at 4 °C in OWB with gentle agitation. The organoids were washed three times, 2 h per wash, before overnight incubation with secondary antibodies at 4 °C. The three-wash procedure was repeated after secondary staining. Samples were pelleted at 70 × g for 2 min at 4 °C and transferred to a fructose-glycerol clearing solution for 20 min at room temperature. Cleared organoids were mounted within a PAP-pen boundary on glass slides. Double-sided tape was placed between the slide and coverslip to maintain space for the three-dimensional samples.

Images were acquired on a BC43 Spinning Disk Confocal Microscope (Oxford Andor). Fiji 1.53 was used for image measurements. Mean fluorescence intensity and the fraction of Ki67-positive cells were calculated from independent cultures.

### Cell growth and senescence

Cell growth was recorded at the experimental time points indicated in the figures. Senescence was assessed with the Senescence β-Galactosidase Staining Kit (C0602; Beyotime Biotechnology, Shanghai, China). SA-β-gal-positive cells were identified by their blue staining and photographed with an inverted fluorescence microscope (Nikon Tie, Nikon).

### Neutral lipid and cholesterol assays

For the oxygen-response assay, organoids were exposed to 21% or 5% O_2_ for 12 h. Neutral lipid content was then visualized with LD540. Organoids were fixed in 4% PFA for 15 min, rinsed in PBS, and treated for 30 min at 4 °C with 0.1% digitonin plus 2% BSA. A 15-min block in OWB at 4 °C was performed next. LD540 and Hoechst were diluted in OWB and applied at 4 °C overnight. After staining, the samples were rinsed, mounted, and imaged on the BC43 Spinning Disk Confocal Microscope (Oxford Andor).

Cell viability was measured in parallel with the oxygen experiment. For the nutrient-response study, organoids received glucose (10 mM), insulin (2 μg/mL), or the two in combination. The glucose concentration was selected to represent a nutrient-rich condition relevant to postprandial glucose exposure in rodents. The insulin concentration was used as an experimentally effective stimulation to robustly activate insulin-responsive metabolic signaling *in vitro* and was not intended to reproduce physiological circulating or portal insulin concentrations. Filipin staining was used to visualize free cholesterol. Total cholesterol was measured separately and expressed after normalization to cell number.

### Albumin secretion and urea assay

Albumin secretion was tested at the selected maturation time points. Each condition contained five organoids, with 3 × 10^4^ starting cells per organoid. Organoids were cultured in 100 μL LiverSup medium for 24 h before supernatants were harvested. Freshly isolated primary hepatocytes (PHCs) were cultured in parallel, and their conditioned supernatants were collected as the primary-hepatocyte reference. Mouse albumin was quantified with an ELISA kit (E-EL-M3032-96T, Elabscience) following the kit procedure. The final signal was read at 450 nm, and albumin values were obtained from the assay standard curve.

Urea formation was evaluated in organoids after 4, 8, or 16 days in HMM. At each stage, five organoids containing 3 × 10^4^ starting cells each were exposed to 10 mM ammonium chloride for 18 h. Culture supernatants were then collected. Conditioned supernatants from freshly isolated PHCs were collected in parallel and used as the primary-hepatocyte reference for urea measurement. A 10-μL aliquot from each sample was processed with urea detection reagent (C013-1-1, Nanjing JianCheng Bioengineering Institute). After 15 min in a boiling-water bath, the colorimetric signal was measured at 520 nm.

### RNA sequencing and downstream analysis

Four sample groups were analyzed by bulk RNA sequencing: freshly isolated primary hepatocytes (PHC), passage-2 proliferating hepatocytes (P2), day-8 organoids (OG-D8), and day-16 organoids (OG-D16), with three biological replicates per group. Samples were preserved in TRIzol at −80 °C and transported on dry ice for RNA processing and sequencing at Berry Genomics (Beijing, China). Total RNA quantity and purity were initially assessed using a NanoDrop 2000 spectrophotometer, and RNA integrity was further evaluated using an Agilent 2100 Bioanalyzer with the RNA 6000 Nano kit. Eukaryotic mRNA-seq libraries were subsequently prepared for bulk transcriptome sequencing. The resulting sample-level expression matrix was used for principal-component analysis, hierarchical clustering, differential-expression analysis, pathway heatmaps, gene-set enrichment analysis, and overlap analysis of differentially expressed genes. Differential expression analysis was performed using DESeq2. Gene Ontology (GO) and Kyoto Encyclopedia of Genes and Genomes (KEGG) pathway enrichment analyses were performed using the clusterProfiler package, with mouse gene annotation provided by org.Mm.eg.db. GO enrichment analysis focused on Biological Process (BP) terms, and KEGG enrichment analysis used the *Mus musculus* database (organism = “mmu”). Gene-set enrichment analysis (GSEA) was performed using the gseGO function in clusterProfiler based on genes ranked by DESeq2-derived log2 fold changes, using GO Biological Process gene sets.

### Cell freezing and recovery

Expanded cultures were cryopreserved at the passages indicated in the experiments. Cells were rinsed with PBS, harvested by centrifugation, and suspended in Cebrary® Cell Freezing Medium for Organoids (Yeasen). Aliquots were transferred to cryovials and cooled to −80 °C at about 1 °C per minute. After 24 h, the vials were transferred to liquid nitrogen for storage. Thawed cells were recovered in either 1% FBS-containing medium or TEM. Growth was monitored for 4 days after recovery, and the cells were then tested for organoid formation and liver-marker expression.

### Statistics

GraphPad Prism 10.4.2 was used for statistical testing. Results are reported as mean ± SEM, and each plotted point represents an independent biological or culture replicate. Ordinary one-way ANOVA was applied to datasets with one experimental factor. Ordinary two-way ANOVA was applied when two factors were tested. The analysis used for each figure is stated in its legend. A P value below 0.05 was treated as statistically significant. Significance labels were defined as follows: ns, not significant; *P < 0.05; **P < 0.01; ***P < 0.001; and ****P < 0.0001.

## Results

### A two-stage culture system supports mouse hepatocyte-derived organoid formation

We used separate steps for cell expansion and organoid maturation (Figure 1A). Freshly isolated hepatocyte-derived cells were first grown in two-dimensional culture with TEM. The expanded cells were then moved to low-attachment plates and cultured in HMM for three-dimensional maturation. This approach allowed us to expand the cells before starting the maturation step.

**FIGURE 1:**
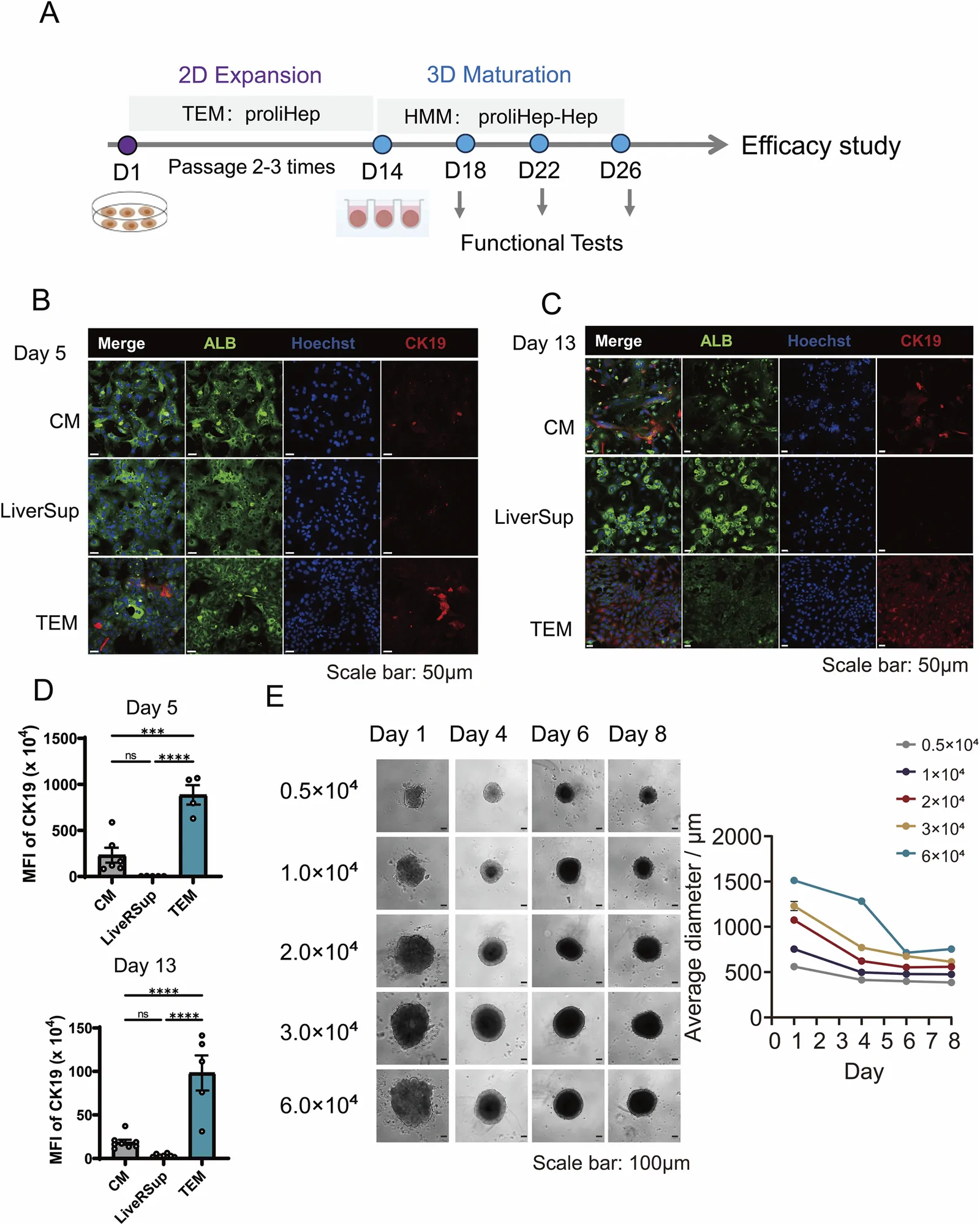
Two-stage expansion and three-dimensional maturation of mouse hepatocyte-derived organoids. **(A)** Timeline of cell expansion in TEM, organoid maturation in HMM, and functional tests. **(B,C)** ALB, CK19, and Hoechst staining in cells cultured in CM, LiverSup, or TEM on days 5 and 13. **(D)** CK19 mean fluorescence intensity on days 5 and 13. **(E)** Bright-field images of spheroids formed from 0.5 × 10^4^ to 6.0 × 10^4^ cells per well on days 1, 4, 6, and 8, with average spheroid diameter. Scale bars are shown in the panels. Data are mean ± SEM. Each dot is an independent culture replicate. Data in **(D)** were analyzed by ordinary one-way ANOVA, and spheroid diameter in **(E)** was analyzed by ordinary two-way ANOVA. ns, not significant; ***P < 0.001; ****P < 0.0001.

ALB was detected in cells grown in CM, LiverSup, and TEM. CK19 staining was much stronger in TEM on both day 5 and day 13 (Figures 1B–D). The cells kept a liver marker and gained a more proliferative, progenitor-like feature. This state was maintained during TEM culture and the cells could then be used for organoid maturation.

Expanded cells formed spheroids in low-attachment plates without shaking or other mechanical force (Figure 1E). The spheroids became smaller during the first few days as the loose cell aggregates compacted. Their size then became more stable. Starting cell number affected final spheroid size. Low cell numbers produced small spheroids, and the highest cell number produced large spheroids that were harder to image and handle. We selected 3 × 10^4^ cells per well because this condition gave uniform and intact organoids.

### TEM supports cell expansion and reduces senescence

We compared cell growth in CM, LiverSup, and TEM during serial culture. Cell density and growth gradually decreased in CM and LiverSup. Cells in TEM continued to reach a density suitable for passage and could be cultured from P0 to later passages (Figure 2A). The cells also kept an adherent epithelial-like shape.

**FIGURE 2:**
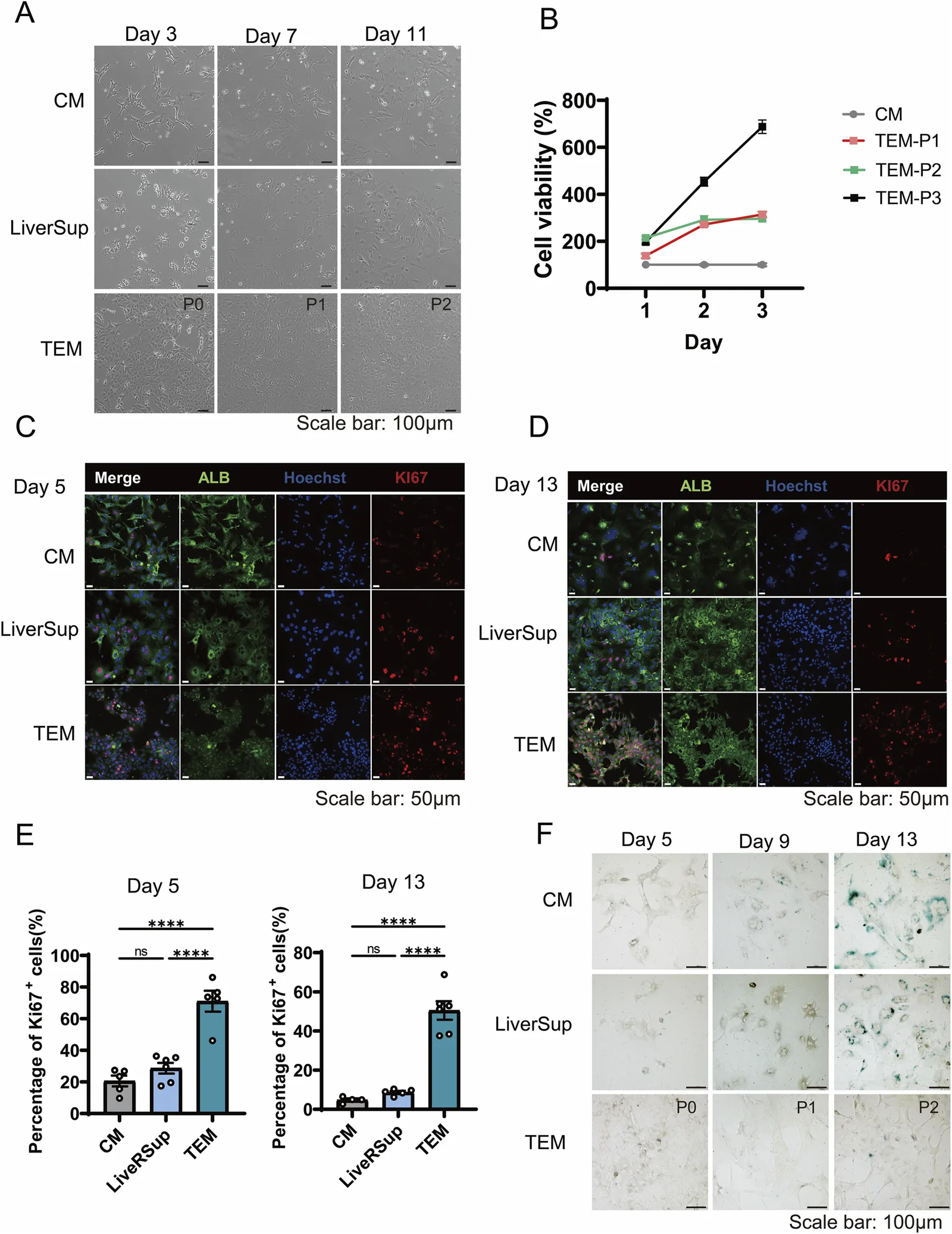
TEM supports cell growth and reduces culture-associated senescence. **(A)** Bright-field images of cells grown in CM, LiverSup, or TEM at the indicated days and passages. **(B)** Relative cell viability or growth of TEM cultures at P1, P2, and P3 compared with CM. **(C,D)** ALB, Ki67, and Hoechst staining on days 5 and 13. **(E)** Percentage of Ki67-positive cells on days 5 and 13. **(F)** SA-β-gal staining in CM, LiverSup, and TEM cultures at the indicated days and passages. Scale bars are shown in the panels. Data are mean ± SEM. Each dot is an independent culture replicate. Data in **(B)** were analyzed by ordinary two-way ANOVA, and data in **(E)** were analyzed by ordinary one-way ANOVA. ns, not significant; ****P < 0.0001.

The growth assay showed little net increase in CM, and TEM cultures increased over time after passage (Figure 2B). ALB remained detectable in the expanded cells. Ki67-positive cells increased in TEM, and the percentage was higher than in CM or LiverSup on both day 5 and day 13 (Figures 2C–E). These data show that TEM kept more cells in an active growth state without loss of ALB expression.

We also measured senescence-associated β-galactosidase activity. Blue-positive cells increased over time in CM and LiverSup, but fewer positive cells were seen in TEM during passage (Figure 2F). TEM supported continued cell growth and reduced the senescence seen in the other culture conditions.

### Three-dimensional maturation partially recovers hepatic markers and metabolic programs

We examined whether expanded cells regained liver features after three-dimensional maturation. The organoids formed compact epithelial structures and expressed ALB and E-cadherin (Figures 3A–C). CK19 was present in some cells, which shows that part of the hepatobiliary or progenitor-like state remained. CYP2E1 was also detected in ALB-positive organoids. This showed that the three-dimensional cultures gained a more differentiated liver phenotype.

**FIGURE 3:**
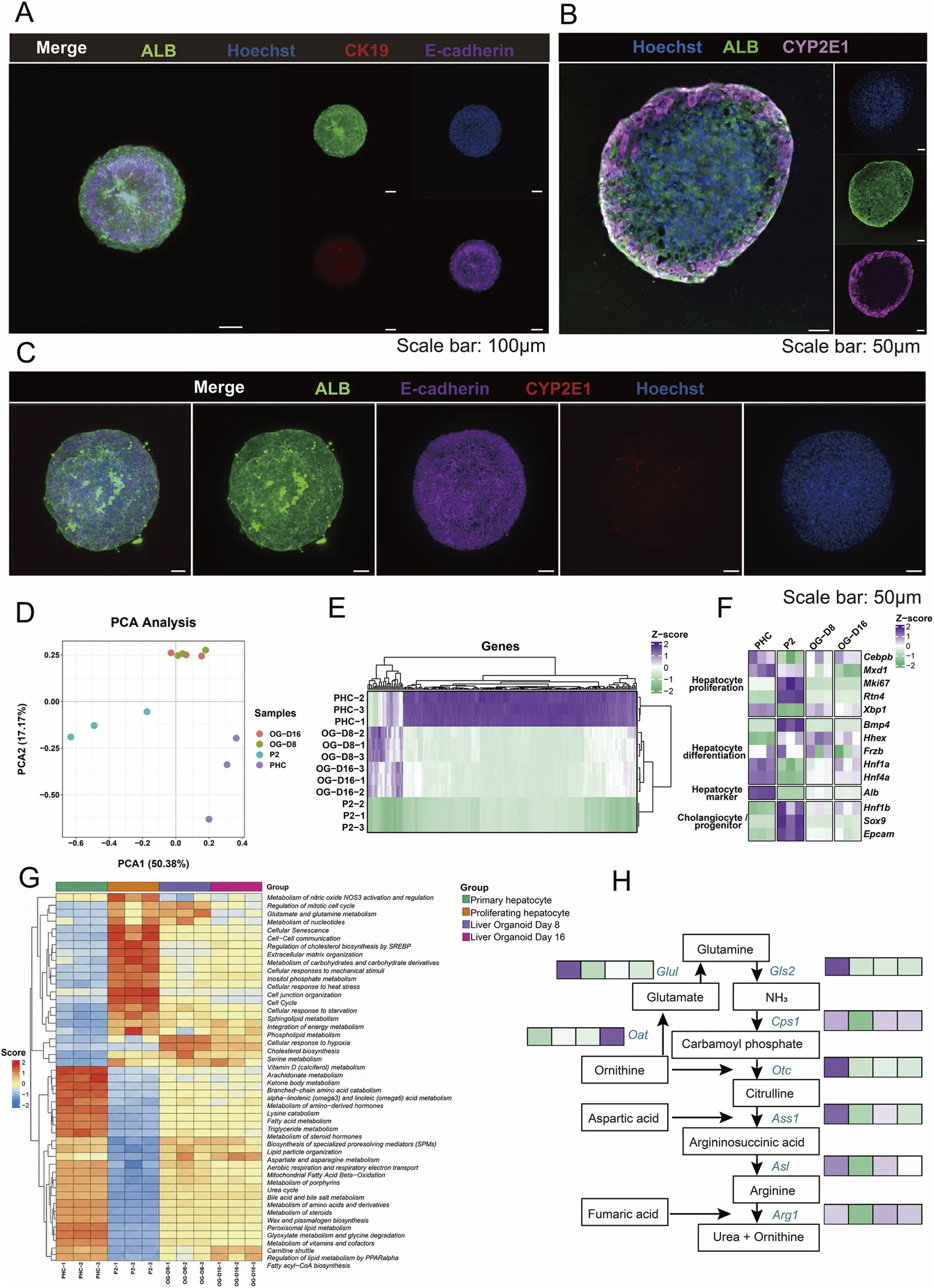
Hepatic markers and RNA profiles during 3D partial hepatic maturation. **(A)** ALB, CK19, E-cadherin, and Hoechst staining in mouse hepatocyte-derived organoids. **(B)** ALB, CYP2E1, and Hoechst staining. **(C)** ALB, E-cadherin, CYP2E1, and Hoechst staining. **(D)** PCA of primary hepatocytes (PHC), passage-2 proliferating hepatocytes (P2), day-8 organoids (OG-D8), and day-16 organoids (OG-D16). **(E)** Heatmap of differentially expressed genes in the four groups. **(F)** Expression heatmap of selected genes associated with hepatocyte proliferation, hepatocyte differentiation, hepatocyte identity, and biliary/progenitor-associated identity across PHC, P2, OG-D8, and OG-D16. Values represent row-scaled Z-scores of group-mean bulk RNA-seq expression. **(G)** Heatmap of selected biological pathways in PHC, P2, OG-D8, and OG-D16. **(H)** Urea-cycle gene expression across the four groups, including *Glul, Gls2, Cps1, Otc, Ass1, Asl, Arg1*, and *Oat.* Scale bars are shown in the panels.

We then compared the RNA profiles of PHC, P2, OG-D8, and OG-D16. PCA clearly separated PHC from P2 (Figure 3D), showing that cell expansion caused large changes in gene expression. PC1 explained 50.38% of the variance and PC2 explained 17.17%. OG-D8 and OG-D16 occupied a similar transcriptional space and remained distinct from both P2 and PHC. Together, these patterns indicate a transition away from the proliferative P2 state during 3D culture without complete restoration of the primary hepatocyte transcriptional profile.

The differentially expressed gene heatmap further distinguished the four states (Figure 3E). Biological replicates from the same condition clustered together. PHC and P2 showed markedly different expression patterns, with many PHC-enriched genes reduced in P2 and a distinct set of genes increased in the proliferative P2 state. Both OG-D8 and OG-D16 shifted away from the P2 pattern and partially recovered PHC-associated gene expression. These results indicate partial transcriptional recovery during 3D culture rather than complete restoration of the primary hepatocyte state.

To further characterize the cellular state of the expanded population, we examined selected markers associated with hepatocyte proliferation, differentiation, and biliary/progenitor identity (Figure 3F). P2 cells showed increased expression of proliferation-associated genes, including *Mki67* and *Rtn4*, together with marked induction of the biliary/progenitor-associated genes *Hnf1b, Sox9,* and *Epcam*. In contrast, several hepatocyte differentiation-associated genes, including *Hnf1a* and *Hnf4a*, were reduced relative to PHCs, although *Alb* remained detectable. Following transfer to 3D culture, the proliferation and biliary/progenitor-associated signatures decreased, whereas several hepatocyte differentiation-associated genes partially recovered. These results indicate that TEM expansion is associated with a transient proliferative and progenitor-like transcriptional state that is partially reversed during subsequent 3D hepatic maturation.

The pathway heatmap showed the same overall change (Figure 3G). P2 cells had strong cell-cycle, DNA-replication, growth, and stress programs, and many liver metabolic pathways were lower. In the organoids, these growth-related programs decreased. Pathways for cholesterol synthesis, steroid metabolism, bile-acid metabolism, fatty-acid oxidation, oxidative metabolism, amino-acid metabolism, and xenobiotic processing increased. Several of these changes were stronger in OG-D16 than in OG-D8.

We also examined genes in the urea cycle and ammonia metabolism (Figure 3H). Expansion changed the expression of *Glul, Gls2, Cps1, Otc, Ass1, Asl, Arg1*, and *Oat*. Three-dimensional culture restored part of this gene pattern, and the changes differed between OG-D8 and OG-D16. The organoids regained parts of the gene program needed for hepatocyte nitrogen metabolism.

GSEA showed clear differences between the expansion and maturation stages (Figures 4A–C). In P2 versus PHC, genes increased in P2 were enriched for epithelial tube formation and the G2/M transition of the mitotic cell cycle (Figure 4A). In OG-D8 versus P2, genes increased in OG-D8 were enriched for cholesterol biosynthesis and the urea cycle (Figure 4B). In OG-D16 versus P2, genes increased in OG-D16 were enriched for steroid biosynthesis and bile-acid biosynthesis (Figure 4C). These comparisons show that liver metabolic programs returned as the cells moved from the expansion stage to partial hepatic maturation.

**FIGURE 4:**
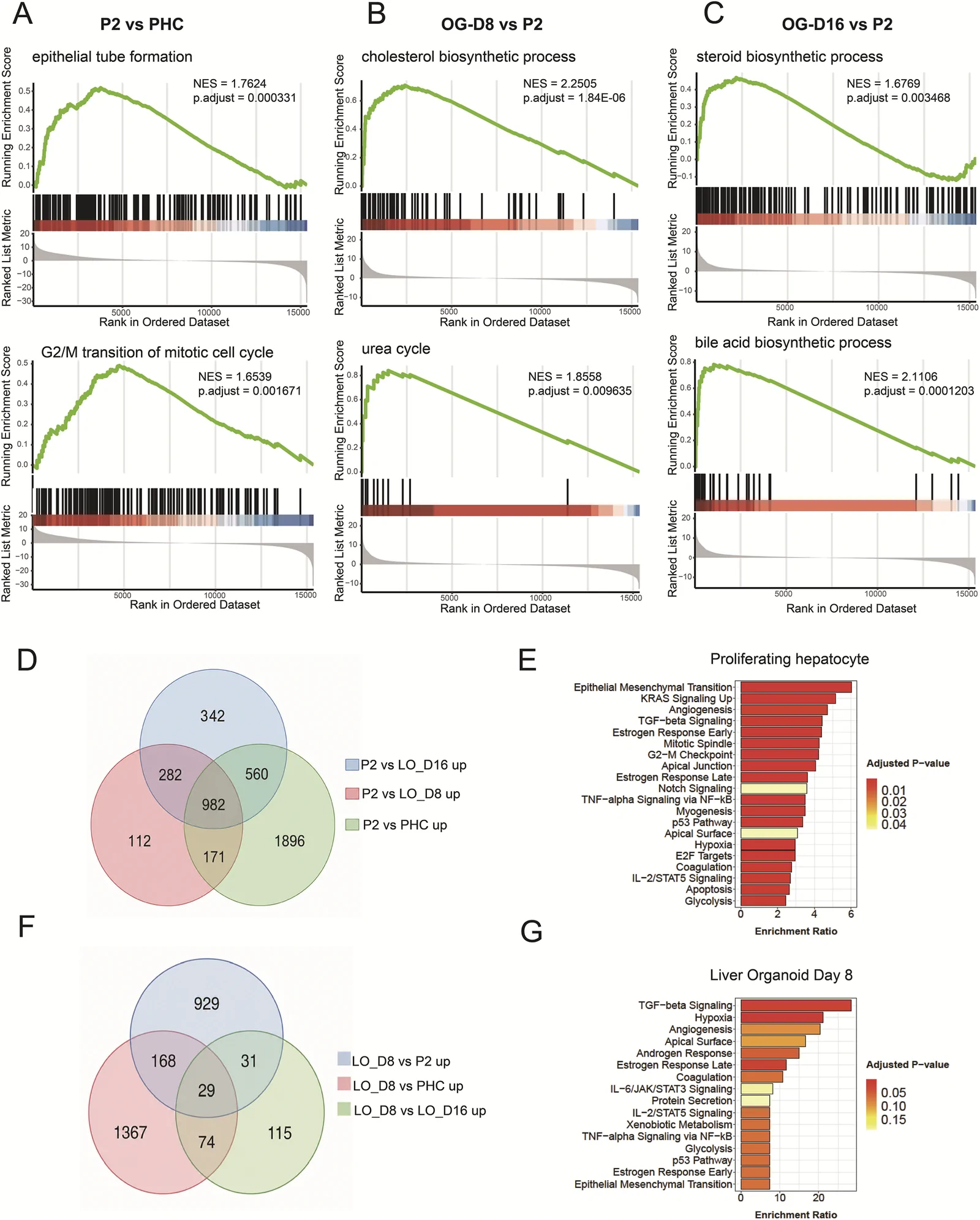
Gene programs linked to cell expansion and 3D partial hepatic maturation. **(A)** GSEA for genes increased in P2 versus PHC, showing epithelial tube formation and G2/M transition of the mitotic cell cycle. **(B)** GSEA for genes increased in OG-D8 versus P2, showing cholesterol biosynthesis and the urea cycle. **(C)** GSEA for genes increased in OG-D16 versus P2, showing steroid biosynthesis and bile-acid biosynthesis. **(D)** Overlap of genes increased in P2 versus OG-D16, OG-D8, and PHC. **(E)** Pathway analysis of the shared P2 gene set. **(F)** Overlap of genes increased in OG-D8 versus P2, PHC, and OG-D16. **(G)** Pathway analysis of the shared OG-D8 gene set. Bar colors show adjusted P values.

A total of 982 genes were increased in P2 compared with PHC, OG-D8, and OG-D16 (Figure 4D). These shared P2 genes were enriched for mitotic spindle organization, G2/M checkpoint control, KRAS signaling, TGF-β signaling, epithelial-mesenchymal transition, angiogenesis, growth-factor responses, and glycolysis (Figure 4E). The P2 state was mainly linked to cell growth and tissue remodeling rather than mature liver metabolism.

We found 29 genes that were increased in OG-D8 compared with P2, PHC, and OG-D16 (Figure 4F). These genes were linked to TGF-β signaling, hypoxia responses, apical-surface organization, cytokine signaling, protein secretion, xenobiotic metabolism, and glycolysis (Figure 4G). This pattern is consistent with an early adjustment to three-dimensional culture. By day 16, this early response was lower and liver metabolic pathways were more evident. Figures 3, 4 show a change from the highly proliferative P2 state to an early organoid state and then to the OG-D16 state with stronger hepatic metabolic programs.

### Mouse hepatocyte-derived organoids respond to oxygen, nutrients, and zonation-related signals

The RNA data showed recovery of several liver metabolic pathways, so we tested the organoids with defined environmental changes. At 5% O_2_, LD540 fluorescence was higher than at 21% O_2_ (Figure 5A), showing more neutral lipid accumulation. Cell viability did not change between the two oxygen conditions (Figure 5B). The increase in lipid staining was not caused by a loss of cell viability.

**FIGURE 5:**
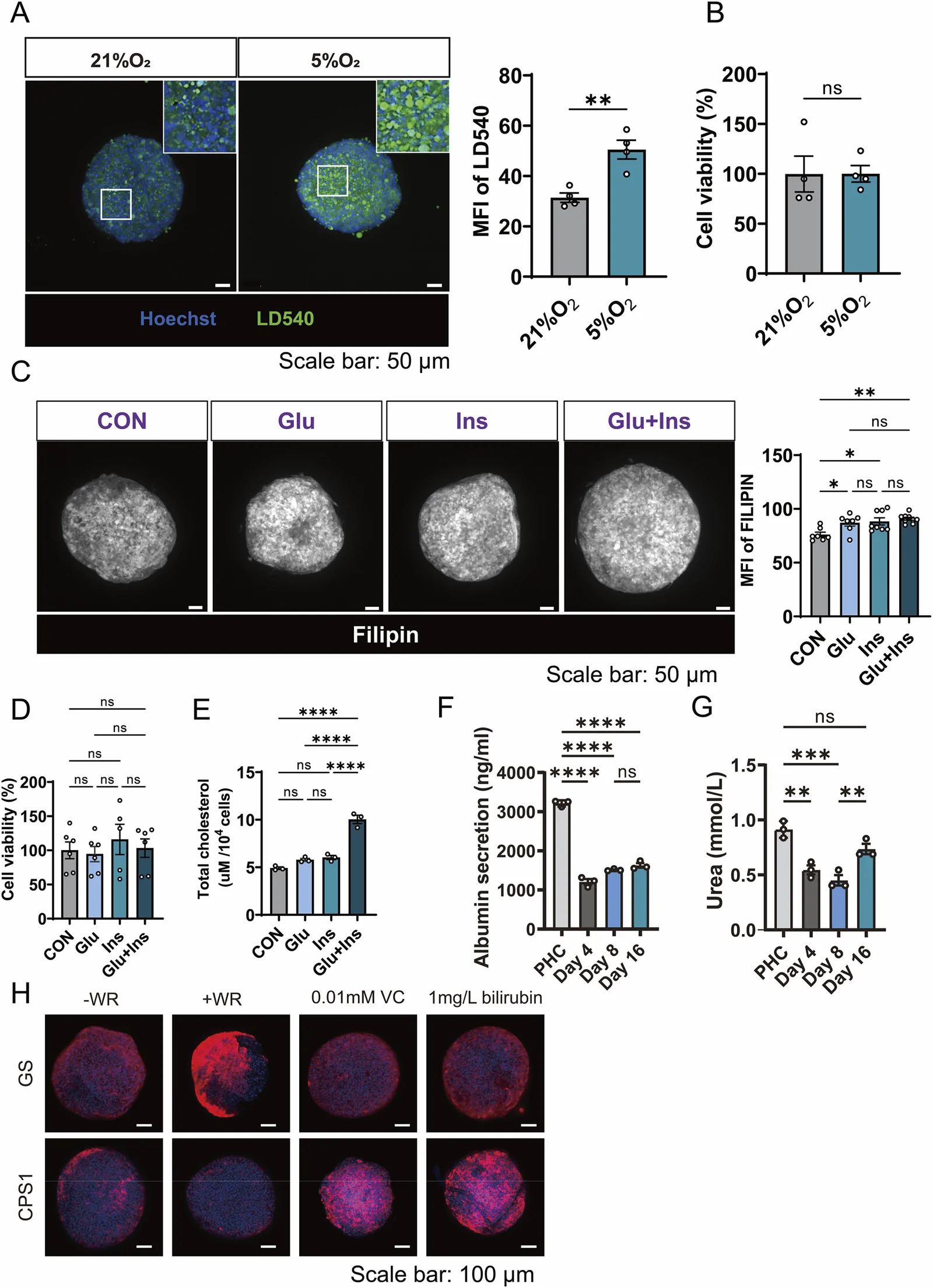
Metabolic functions and environmental responses of mouse hepatocyte-derived organoids. **(A)** LD540 staining and mean fluorescence intensity in organoids cultured at 21% or 5% O_2_. Hoechst marks nuclei. **(B)** Cell viability under 21% and 5% O_2_. **(C)** Filipin staining and mean fluorescence intensity in control organoids and organoids treated with glucose (10 mM), insulin (2 μg/mL), or glucose plus insulin. **(D)** Cell viability after nutrient treatment. **(E)** Total cholesterol normalized to cell number. **(F)** Albumin secretion by freshly isolated primary hepatocytes (PHC) and organoids after 4, 8, or 16 days of 3D culture. **(G)** Urea production by freshly isolated PHCs and organoids after 4, 8, or 16 days of 3D culture. **(H)** GS and CPS1 staining in organoids cultured without Wnt/R-spondin (-WR), with Wnt/R-spondin (+WR; 5 ng/mL each), with ascorbic acid (0.01 mM), or with bilirubin (1 mg/L). Scale bars are shown in the panels. Data are mean ± SEM. Each dot represents an independent biological or culture replicate. Data in **(A–G)** were analyzed by ordinary one-way ANOVA. ns, not significant; *P < 0.05; **P < 0.01; ***P < 0.001; ****P < 0.0001.

We tested cholesterol metabolism with glucose and insulin. Glucose or insulin alone caused a small increase in filipin staining, and glucose plus insulin gave the strongest free-cholesterol signal (Figure 5C). Cell viability was similar among the groups (Figure 5D). Total cholesterol showed the same pattern, with the highest value after glucose plus insulin treatment (Figure 5E). These findings demonstrate that the organoids retained responsiveness of cholesterol metabolism to glucose and insulin stimulation.

Albumin secretion and urea production were directly compared with freshly isolated primary hepatocytes (PHCs) to assess functional recovery during 3D culture. Albumin secretion remained significantly lower than that of PHCs at all organoid stages, although it increased during 3D culture and reached similar levels at days 8 and 16 (Figure 5F). In contrast, urea production showed a more pronounced recovery (Figure 5G). Urea production was reduced at the earlier organoid stages but increased by day 16, reaching a level that was not significantly different from freshly isolated PHCs. Day-16 organoids also showed higher urea production than the earlier organoid stages. These findings indicate function-specific recovery during 3D culture, with urea production approaching the primary hepatocyte level by day 16, whereas albumin secretion remained only partially restored.

We also tested signals linked to liver zonation. Wnt/R-spondin increased glutamine synthetase, a pericentral marker, and CPS1 staining was lower. Ascorbic acid and bilirubin increased CPS1, a periportal marker (Figure 5H). These treatment concentrations were selected from previously established hepatocyte-derived organoid studies and were used as pathway-modulating experimental conditions rather than as direct reproductions of *in vivo* zonal concentrations. These treatments do not reproduce the full portal-to-central vein gradient, but they show that the organoids can change in response to zonation-related signals. This feature can be used in later studies of Kupffer cell-hepatocyte interactions under different oxygen and nutrient conditions.

### Expanded cells retain organoid-forming ability after cryopreservation

We tested whether the expanded cells could be passaged, frozen, and used again without a new primary hepatocyte isolation. Cells kept an adherent proliferative shape from P2 to P5 and could be passaged during the 17-day observation period (Figure 6A). Cell density increased again after each passage, showing continued growth during serial culture.

**FIGURE 6:**
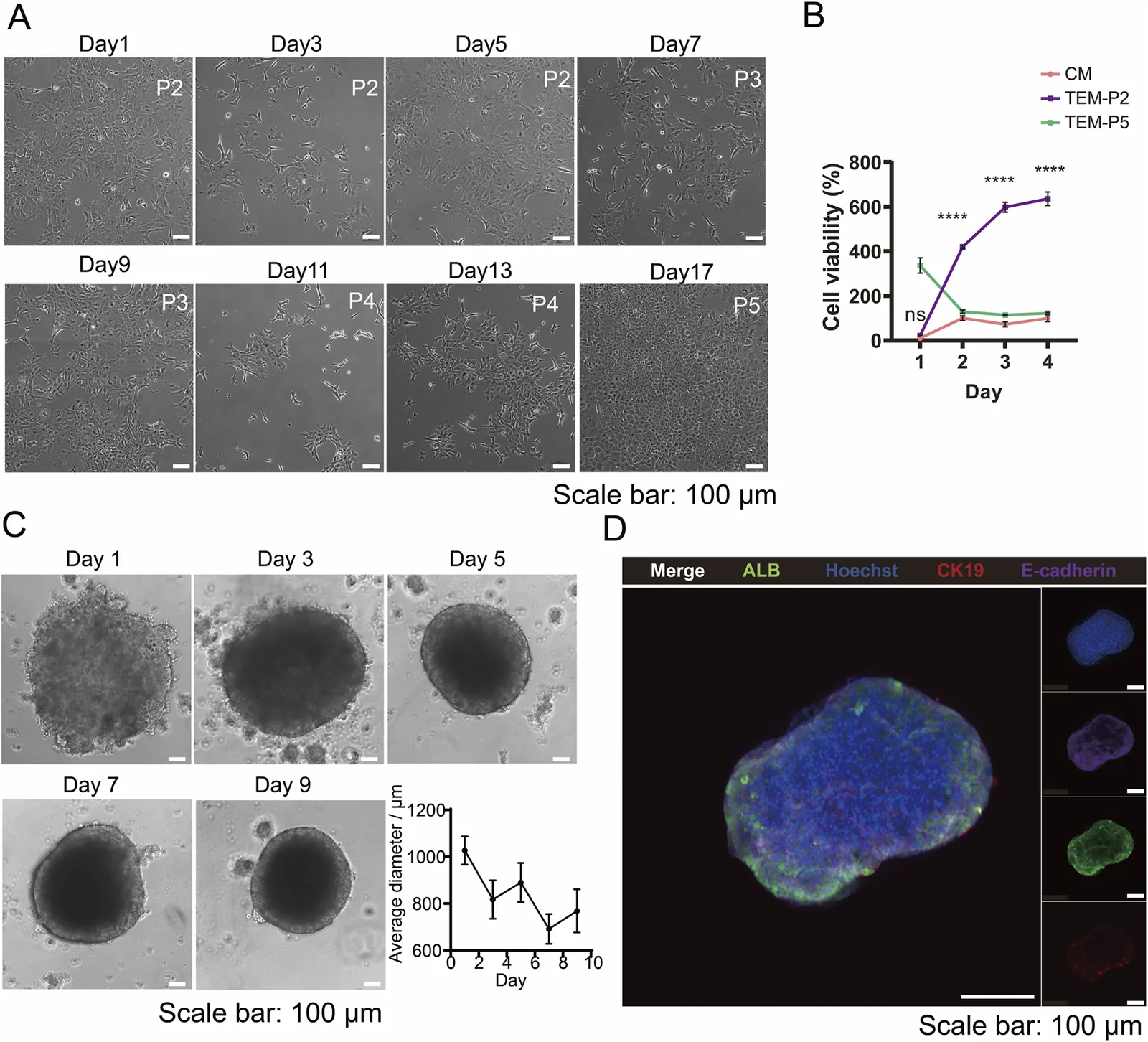
Cell expansion and organoid formation after cryopreservation. **(A)** Bright-field images of hepatocyte-derived cells during serial expansion from P2 to P5. **(B)** Relative post-thaw cell viability of passage-2 and passage-5 cells cultured in 1% FBS-containing medium or TEM, assessed by CCK-8 assay. Values are expressed relative to the indicated baseline condition. **(C)** Bright-field images of organoid formation from day 1 to day 9, with average spheroid diameter. **(D)** Hoechst, ALB, E-cadherin, and CK19 staining in recovered organoids. Scale bars are shown in the panels. Data are mean ± SEM. Data in **(B)** were analyzed by ordinary two-way ANOVA, and spheroid diameter in **(C)** was analyzed by ordinary one-way ANOVA. ns, not significant; *P < 0.05; **P < 0.01; ***P < 0.001; ****P < 0.0001.

Post-thaw recovery, assessed by relative CCK-8 metabolic activity, differed according to passage number and recovery condition (Figure 6B). P2 cells recovered in the 1% FBS-containing CM condition showed the largest increase in CCK-8 signal over 4 days, whereas P5 cells showed little increase under either recovery condition. These findings indicate greater post-thaw metabolic recovery in early-passage cells under the current freezing protocol. Because CCK-8 provides an indirect measure of metabolic activity rather than a direct measurement of cell survival, these data should not be interpreted as quantitative evidence of post-thaw viability.

Recovered early-passage cells could still form organoids. The new aggregates compacted from day 1 to day 3 and then kept a mainly spherical shape through day 9 (Figure 6C). The change in average diameter during the first days matched the visible compaction and reorganization of the aggregates.

Post-thaw organoids expressed ALB and epithelial or hepatobiliary-associated markers (Figure 6D). This shows that early-passage cells kept organoid-forming ability and liver lineage features after freezing. P5 recovery was weak, so later-passage banking may require changes in freezing medium, passage timing, or post-thaw culture conditions.

## Discussion

This study established a two-stage culture strategy that separates hepatocyte expansion from subsequent 3D partial hepatic maturation. TEM generated sufficient cells for repeated experiments, whereas transfer to HMM promoted recovery of several hepatic markers, metabolic programs, and functional properties. This approach addresses a common challenge in hepatocyte culture, in which proliferative expansion is frequently accompanied by loss of differentiated hepatic functions (Baker and Chen, 2012; Booth et al., 2012; Huch and Koo, 2015; Huch et al., 2013; Igarashi et al., 2025).

The technical scope of this system differs from that of previously established hepatocyte organoid models. Earlier mouse primary hepatocyte organoid systems demonstrated robust long-term expansion in three-dimensional extracellular-matrix-based cultures, whereas recent mouse assembloid approaches have incorporated cholangiocytes and mesenchymal cells to reconstruct aspects of periportal architecture and bile drainage (Dowbaj et al., 2025). Recent human systems have further achieved advanced metabolic functions or spatially organized multi-zonal phenotype (Igarashi et al., 2025; Reza et al., 2025). In contrast, the present study was designed as a simpler hepatocyte-focused platform in which the expansion and 3D hepatic-recovery phases are physically and temporally separated. This configuration allows the proliferative state and the subsequent partially matured state to be examined independently and facilitates controlled testing of metabolic and environmental cues. Thus, the main technical value of the model lies in its two-stage experimental design and accessibility for mechanistic perturbation studies, rather than in reproducing the full cellular complexity, long-term expansion capacity, or spatial organization achieved by more advanced liver organoid systems.

During TEM expansion, the cells retained ALB expression, showed increased Ki67 expression, and exhibited reduced senescence, while CK19 expression also increased. The additional transcriptional marker analysis further supported a shift away from the fully differentiated hepatocyte state. P2 cells showed increased expression of proliferation-associated genes together with induction of the biliary/progenitor-associated markers *Hnf1b, Sox9,* and *Epcam*, whereas several hepatocyte differentiation-associated genes were reduced. After transfer to 3D culture, the proliferation and progenitor-associated signatures decreased and hepatocyte differentiation programs partially recovered. These findings are consistent with a transient proliferative state with hepatobiliary/progenitor-associated features during expansion, followed by partial redifferentiation during 3D culture.

However, the present bulk RNA-seq data cannot determine whether hepatocyte and progenitor markers are co-expressed within the same cells or arise from distinct subpopulations. Therefore, the current data do not allow us to distinguish conclusively between dedifferentiation of mature hepatocytes and selective enrichment of a pre-existing progenitor-like population. Similarly, expression of *Sox9, Epcam,* and *Hnf1b* alone does not establish true bipotent differentiation potential. Lineage-tracing studies, single-cell transcriptomic analysis, and functional hepatocyte-versus-biliary differentiation assays will be required to resolve these possibilities more precisely.

Bulk transcriptomic analysis further demonstrated substantial differences between P2 cells and freshly isolated primary hepatocytes (PHCs), consistent with the phenotypic remodeling associated with expansion. Following transfer to HMM, both OG-D8 and OG-D16 shifted away from the proliferative P2 state and partially recovered hepatocyte-associated transcriptional programs. Nevertheless, neither organoid stage fully reproduced the transcriptional profile of fresh PHCs. Pairwise GSEA suggested that this recovery occurred in a stepwise and function-specific manner. P2 versus PHC was characterized predominantly by epithelial remodeling and G2/M-associated activity. OG-D8 versus P2 showed enrichment of cholesterol biosynthesis and the urea cycle, whereas OG-D16 versus P2 showed enrichment of steroid and bile-acid biosynthesis. These results suggest that different hepatic programs recover with distinct temporal patterns during 3D culture. Although OG-D16 showed enrichment of later-stage metabolic pathways, OG-D8 and OG-D16 remained transcriptionally distinct from fresh PHCs, consistent with partial rather than complete hepatic maturation. Similar limitations have been reported in other expandable liver organoid models (Booth et al., 2012; Igarashi et al., 2025; Reza et al., 2025).

Functional assays were broadly consistent with the transcriptomic findings. Direct comparison with freshly isolated PHCs showed that functional recovery differed among hepatic endpoints. Albumin secretion increased during 3D culture but remained significantly below the PHC level even at day 16. In contrast, urea production increased at the later stage of culture and, by day 16, was not significantly different from the PHC control. These findings indicate that individual hepatic functions recover at different rates and further support the interpretation of partial and function-specific hepatic maturation. Nevertheless, albumin secretion and urea production represent only a subset of mature hepatocyte functions and are insufficient on their own to establish complete functional maturation. Other key functions, including cytochrome P450-mediated metabolism, drug metabolism, bile-acid transport, and canalicular activity, were not assessed in the present study and will require further evaluation.

The organoids also retained responsiveness to selected environmental and zonation-associated cues. Reduced oxygen increased lipid-droplet accumulation, whereas glucose plus insulin enhanced cholesterol accumulation. Wnt/R-spondin treatment increased GS expression, while ascorbic acid or bilirubin increased CPS1 expression. These findings indicate that the organoids can respond to environmental signals associated with metabolic heterogeneity across the liver lobule (Ben-Moshe and Itzkovitz, 2019; Halpern et al., 2017; Martini et al., 2023; Samarah et al., 2025; Reza et al., 2025). The experimental concentrations used here differed in their physiological relevance. Glucose at 10 mM was selected to represent a nutrient-rich condition relevant to postprandial glucose exposure in rodents. In contrast, insulin at 2 μg/mL was used as an experimentally effective stimulation to activate insulin-responsive metabolic signaling and should not be interpreted as a physiological circulating insulin concentration. The Wnt/R-spondin, bilirubin, and ascorbic acid conditions were adopted from previously established liver organoid studies to modulate zonation-associated pathways (Igarashi et al., 2025; Reza et al., 2025). These treatments were therefore intended to assess responsiveness to selected environmental and zonation-associated cues rather than to quantitatively reproduce their endogenous concentrations or the continuous periportal-to-pericentral gradients present *in vivo*. Accordingly, the present model should be considered a system for studying zonation-related responses rather than one that fully recapitulates physiological liver zonation.

The cells could be serially passaged through P5, cryopreserved, recovered, and subsequently used for organoid formation. Post-thaw CCK-8 measurements showed a greater increase in metabolic activity in early-passage cells than in later-passage cells under the current freezing protocol. However, CCK-8 reflects cellular metabolic activity and does not directly quantify live- and dead-cell fractions. Therefore, the present data support differences in post-thaw recovery but do not provide a direct measurement of post-thaw survival. Direct Live/Dead staining and quantitative assessment of dead-cell percentages will be required in future studies to more rigorously evaluate post-thaw survival across passages. The weaker recovery observed at later passages also suggests that passage number may influence banking performance. In addition, systematic measurements of expansion efficiency, cumulative population doublings, phenotypic stability, and genetic stability over extended culture periods will be required to more comprehensively define the long-term expansion capacity of this system.

Several additional limitations should be considered. First, the model is predominantly hepatocyte-derived and does not reproduce the full multicellular liver microenvironment. Kupffer cells, liver sinusoidal endothelial cells, hepatic stellate cells, biliary cells, extracellular-matrix components, and perfusion-derived signals can all influence hepatocyte differentiation, metabolism, and zonation (Booth et al., 2012; Ouchi et al., 2019; Takebe et al., 2013; Dowbaj et al., 2025; Igarashi et al., 2025; Reza et al., 2025). Incorporation of selected non-parenchymal cell populations and more physiologically structured culture conditions may further improve hepatic function and spatial organization. Second, although the present study demonstrates partial recovery of hepatic transcriptional and functional properties, broader mature hepatocyte functions remain to be evaluated. Third, the zonation-related experiments assess responses to individual cues rather than the formation of stable spatially organized zonal states.

Overall, the present findings support a two-stage strategy in which hepatocyte expansion is followed by partial recovery of hepatic identity and function in 3D culture. Rather than fully reproducing mature primary hepatocytes or physiological liver zonation, this model provides an experimentally accessible platform for examining hepatocyte expansion, partial hepatic maturation, metabolic regulation, and responses to selected microenvironmental and zonation-associated signals.

## Conclusion

We established a two-stage mouse hepatocyte-derived organoid culture system that separates hepatocyte expansion from subsequent 3D partial hepatic maturation. The expansion medium supported serial passaging through P5 while retaining hepatocyte-associated features, and early-passage cells retained organoid-forming capacity after cryopreservation and recovery. Following transfer to 3D culture, the organoids partially recovered hepatic transcriptional programs and functional properties. Direct comparison with freshly isolated primary hepatocytes showed that albumin secretion remained below the PHC level, whereas urea production approached the PHC level by day 16, supporting partial and function-specific hepatic maturation. The organoids also retained responsiveness to changes in oxygen, glucose, insulin, and selected zonation-associated signals. Although the organoids did not fully reproduce the mature primary hepatocyte state or physiological liver zonation, this system provides an experimentally accessible *in vitro* platform for investigating hepatocyte expansion, partial hepatic maturation, metabolic regulation, zonation-related responses, and microenvironment-dependent modulation of hepatocyte function.

## Data Availability

The RNA-sequencing data generated in this study have been deposited in the NCBI Gene Expression Omnibus (GEO) under accession number GSE350774 and are publicly available at https://www.ncbi.nlm.nih.gov/geo/query/acc.cgi?acc=GSE350774.Additional data supporting the findings of this study are available from the corresponding author upon reasonable request.
